# Clinical Performance and Trends during the First Two Months of Monkeypox Virus PCR Testing at Two United States Reference Labs

**DOI:** 10.1128/jcm.01371-22

**Published:** 2022-11-21

**Authors:** Nicole A. P. Lieberman, Patrick C. Mathias, Benjamin T. Bradley, Alexander L. Greninger

**Affiliations:** a Department of Laboratory Medicine and Pathology, University of Washington School of Medicine, Seattle, Washington, USA; b Department of Biomedical Informatics and Medical Education, University of Washington School of Medicine, Seattle, Washington, USA; c Department of Pathology, University of Utah, Salt Lake City, Utah, USA; d ARUP Laboratories, Salt Lake City, Utah, USA; e Vaccine and Infectious Disease Division, Fred Hutchinson Cancer Research Center, Seattle, Washington, USA; Cepheid

**Keywords:** MPXV, monkeypox, multiple swabs, qPCR, viral load

## Abstract

Recently, a sustained human-to-human outbreak of monkeypox virus (MPXV), a member of the *Orthopoxvirus* genus, which includes the etiologic agent of smallpox, has been documented in multiple nonendemic countries, including the United States. Prior to June 2022, testing in the United States was limited to public health labs and the Centers for Disease Control and Prevention. Following recognition of the scope of the outbreak, testing for MPXV has expanded into clinical laboratories. Here, we examine epidemiological characteristics, specimen collection practices, and cycle threshold (Ct) values for 10,019 MPXV PCR tests performed at two reference laboratories. Results from both laboratories support public health data showing a high positivity rate in men (>30%) and those ages 30 to 49 (25 to 35%). The overall positivity rate decreased during the study period but remains elevated (~20%). There was a significant difference in Ct values between laboratories (ARUP 23.9 versus UW 25.4) and collection method (22.8 for dry swab versus 24.4 for VTM), likely reflecting slight differences in specimen processing. When multiple specimens were collected for a single individual, the overall result concordance rate was greater than 95%, with less than 1.5% of individuals receiving three or more tests having a single positive result. Compared to the overall positive cohort, individuals with three or more swabs collected and a single positive result had significantly higher Ct values (22.9 versus 35.0). Intriguingly, individuals aged 50 to 59 years old had a significantly different viral load distribution than those found in younger age groups, potentially associated with prior vaccinia virus vaccination. These results provide an early snapshot of testing in the United States during the monkeypox virus outbreak and support restricting the number of swabs collected per individual.

## INTRODUCTION

Monkeypox virus (MPXV) is a member of the *Orthopoxvirus* genus that has caused a recent public health emergency of international concern, infecting more than 55,000 persons around in the world and more 21,000 persons in the United States in 2022 as of September 10, 2022. Orthopoxviruses have not spread widely in humans within the United States for approximately 80 years and vaccinia virus vaccination stopped being routine in the United States 50 years ago, leading to substantial gaps in immunity, clinician familiarity with orthopoxvirus infections, and diagnostic testing (1). MPXV PCR diagnostic testing on dry lesion swabs in the United States was initially performed using an FDA-authorized orthopoxvirus test run by the Laboratory Response Network (LRN) with confirmatory reflex MPXV-specific testing performed by the Centers for Disease Control and Prevention (CDC) (2, 3). In June/July 2022, testing expanded to clinical microbiology laboratories where FDA-authorized and laboratory developed tests (LDTs) are currently available. These LDTs expanded the range of testable specimen types from the initial offering of the FDA-authorized method (dry swabs) to include viral transport media as well as other alternative specimen types. Molecular targets also vary between LDTs and include conserved *Orthopoxvirus* genus-level targets, probes specific for the West African clade of MPXV, or a multiplexed approach (3, 4). On September 7, 2022, the Secretary of Health and Human Services authorized the emergency use of *in vitro* diagnostics for the detection and/or diagnosis of MPXV infection (5).

As evidenced by the SARS-CoV-2 pandemic, clinical and reference laboratories play a critical role in the response to newly emergent viral diseases in the United States (6). Testing capacities in reference laboratories are often substantially higher than that of the public health system and may provide integrated ordering and reporting of results that can substantially augment detection of viral outbreaks. While qualitative monkeypox virus test results are reported to state health departments and the CDC, aspects of test performance and utility such as cycle threshold (Ct) values or concordance across multiple specimens from the same individual are not readily reported by public health authorities. These data may contain essential information allowing clinical laboratorians to identify emerging viral mutations and steward appropriate testing practices (7, 8). How testing is transitioned from public health laboratories to clinical laboratories at the start of an outbreak remains of considerable interest for American public health and pandemic preparedness. To date, the only published report of MPXV PCR testing data in the United States only described the weekly volumes, positivity rate, and turnaround times from tens of Laboratory Response Network Labs (2). Here, we examined MPXV PCR testing data from more than 10,000 specimens associated with early testing in July and August 2022 from two references laboratories in the western United States. ARUP Laboratories (ARUP) in Salt Lake City, UT, performed 8,625 MPXV PCR tests on 6,014 individuals, and the University of Washington Virology Lab (UW) in Seattle, WA, performed 1,394 tests on 1,218 individuals, for a total of 10,019 results from 7,232 individuals in our data set.

## MATERIALS AND METHODS

### MPXV PCR testing at UW.

Skin swabs were collected in 3-mL viral transport media (VTM). UW performs specimen inactivation and lysis in a biosafety cabinet by adding 100 μL buffer AL (Qiagen) to 100 μL of swab VTM. This combined mixture is used as input to the DNA and Viral NA Small Volume Kit 2.0 (Roche) on the MP96 system for nucleic acid extraction with 100 μL eluate. Each 25 μL PCR consisted of the equivalent of 2.71 μL water, 11.95 μL QuantiTect master mix, 0.1 μL each of 100 μM forward and reverse primers targeting the MPXV F3L gene (9), 0.05 μL of 100 μM probe, 0.063 μL of an EXO external control primer/probe mix, 0.025 μL of UNG enzymes, and 10 μL of extracted DNA per reaction. Each reaction was run on ABI 7500 using 2 min 50°C; 15 min 95°C; 45 cycles of 1 min 94°C and 1 min 60°C and positives were called at Ct < 40.

### MPXV PCR testing at ARUP.

Skin swabs were collected in-3 mL VTM. Presumptive detection of monkeypox virus from clinical samples was performed by a laboratory-developed real-time PCR assay using primer and probes designed by ELITech (Bothell, WA, USA) targeting a conserved region within the polymerase gene common among the Orthopoxvirus family. Samples received on dry swabs were submerged in 500 μL of PBS, vortexed for 15 s, then allowed to sit at room temperature for 1 h before proceeding to extraction. For specimens in VTM and dry swabs following resuspension, 200 μL of sample was eluted into 80 μL on the Chemagic MSMI (PerkinElmer, Waltham, MA, USA) instrument. Following reaction set-up, amplification was performed on the QuantStudio 12K Flex (Thermo Fisher Scientific, Waltham, MA, USA) to determine the cycle threshold (Ct). During the study, the assay was moved to the cobas 6800 (Roche Diagnostics, Indianapolis, IN) platform which includes nucleic acid extraction and amplification. The same primer/probe sequences were used on this platform as the QuantStudio. A method comparison between the two assays demonstrated comparable performance. If Ct was <36 the specimen was reported as positive, if 36 to 40 repeated (including repeat extraction and amplification), and if >40 reported as negative. If amplification was detected (<40) on repeat it was called positive, and if the sample did not amplify (>40) it was called “inconclusive.” Inconclusive results comprised a small minority of samples (*n* = 15, 0.17% of tests) and were excluded from this analysis.

### Ethics, data analysis, and statistics.

An IRB exemption was granted by the University of Utah IRB under ID#: 00158025. The study was approved by the University of Washington Institutional Review board under STUDY00010205. Deidentified data were analyzed in R v4.0.3. Test results from specimen types other than mucocutaneous lesion swabs were removed by filtering on terms, including “serum,” “cerebrospinal,” “breastmilk,” and “blood.” Inhibited, invalid, and inconclusive results, comprising 0.18% of results, were excluded from analysis. When considering replicate samples submitted for the same individuals, samples collected on different days were not considered part of the same collection event and those individuals were excluded from replicate analysis.

## RESULTS

### Descriptive epidemiology of MPXV PCR testing at ARUP and UW.

**FIG 1:**
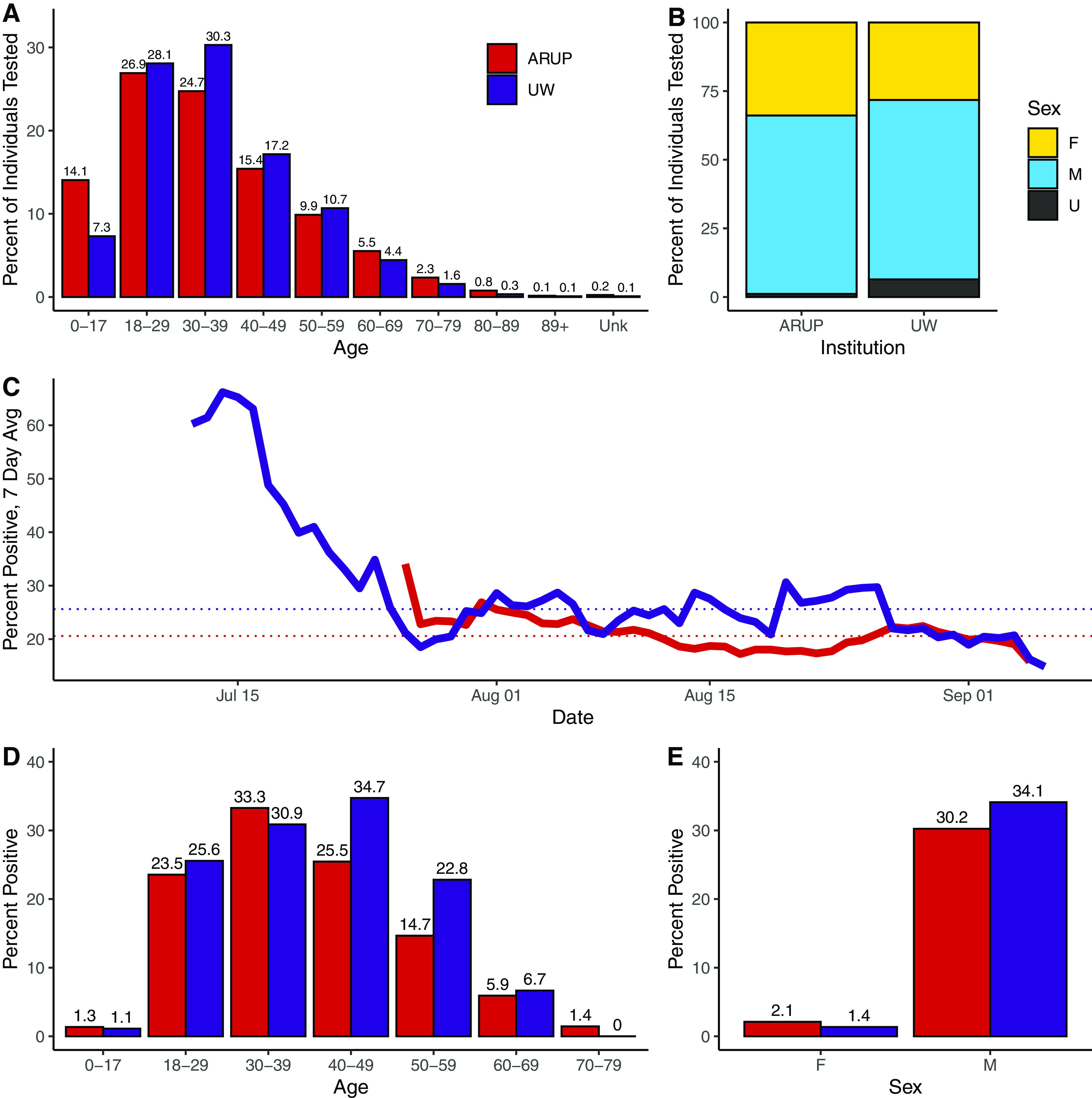
Descriptive epidemiology of individuals tested for MPXV by qPCR at two institutions. (A) Age distribution of individuals tested at ARUP and UW as a percentage of all individuals. (B) Percent of individuals tested, by sex, at ARUP and UW. M = male, F = female, U = unknown/other. (C) Seven-day rolling average of positive MPXV qPCR test results. (D) Percent of tests positive for MPXV by age. (E) Percent of tests positive for MPXV by sex.

We first examined the age and sex distributions of individuals tested at each reference lab. When the age distribution of individuals tested at UW was compared to the age distribution in Seattle from the 2020 United States census data (10), it was not significantly different (*P* = 0.139, chi squared). Median age of all tested individuals was 33 at both institutions, with an age range of 0 to 92 (UW) or 0 to 99 (ARUP). ARUP had a larger proportion of pediatric (age < 18 years old) individuals tested for MPXV than UW did (Fig. 1A). At UW, the median age of males tested was 34 (range 0 to 84) and females was 32 (range 0 to 92). At ARUP, the median age of males tested was 33 (range 0 to 94) and females was 31 (range 0 to 99).

At both UW and ARUP, males comprised about 65% of individuals tested for MPXV (Fig. 1B). Females accounted for a larger share of individuals tested for MPXV at ARUP than at UW (33.9% versus 28.2%, respectively), while UW had more individuals whose sex was unknown (6.4% versus 1.1%). When individuals with unknown sex were excluded, the distribution was significantly different between the institutions (*P* = 0.007, chi squared).

Both ARUP and UW started offering MPXV testing in July of 2022, after which the test positivity rate has decreased (Fig. 1C). Driven in part by much higher positivity shortly after the initiation of testing, the cumulative percent positivity has been higher at UW (25.6% versus 20.6%, dotted lines on Fig. 1C). The highest test positivity rate at UW was seen in the 40 to 49 age group (34.7%), while at ARUP, ages 30 to 39 had the highest rate of positive tests (33.3%) (Fig. 1D). At both UW and ARUP, males had a much higher test positivity rate than females (34.1% versus 1.4% at UW, and 30.3% versus 2.1% at ARUP) (Fig. 1E).

### Comparable viral loads in positive specimens at ARUP and UW.

**FIG 2:**
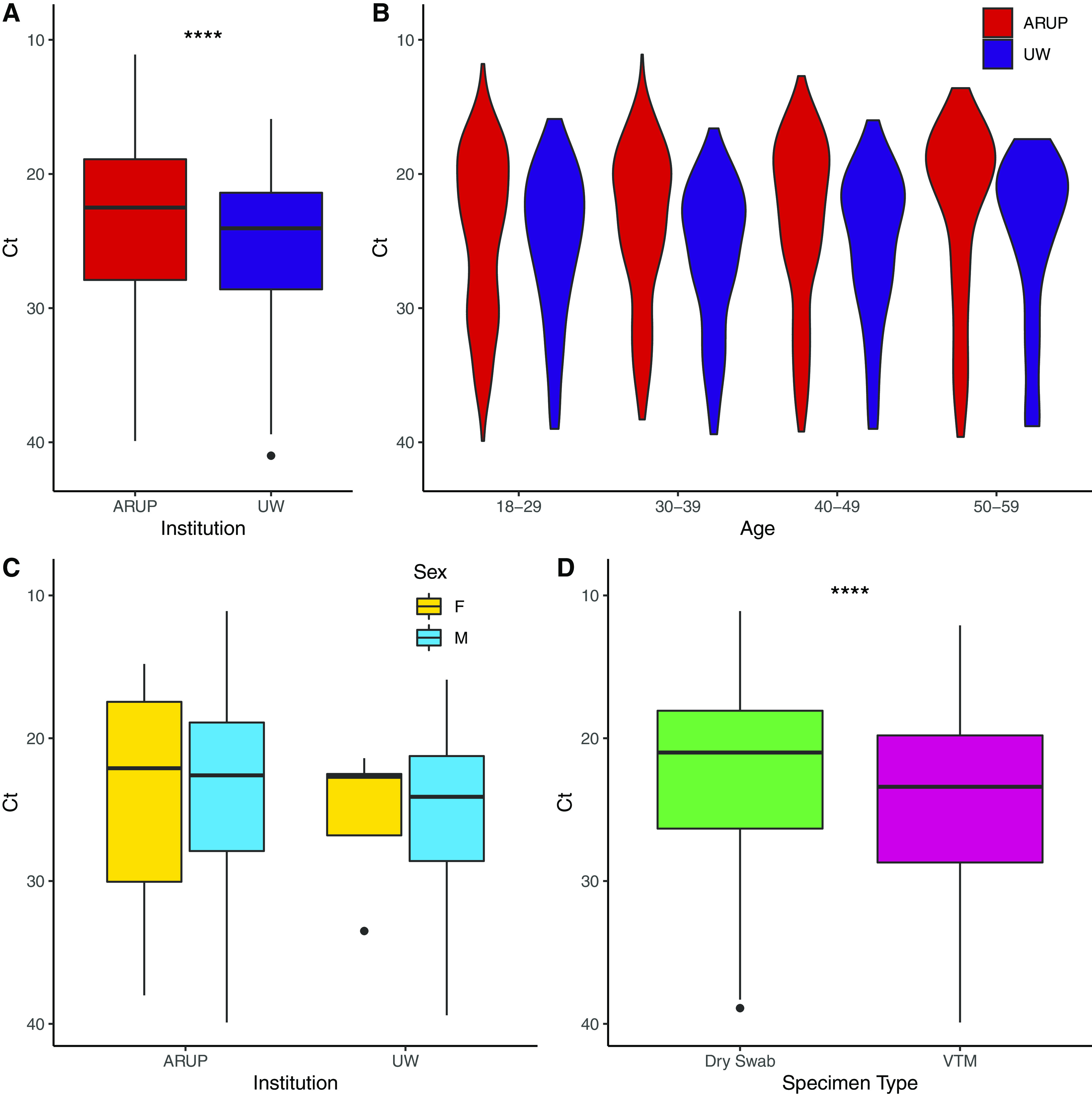
MPXV cycle threshold differences. Median and interquartile range (IQR) are shown in boxplots, with whiskers representing 1.5× IQR. Outliers are shown as individual points. (A) Ct values of positive tests by institution. ****, *P* < 0.0001, *t* test. (B) Violin plot of Ct values of positive tests by age. (C) Ct values of positive tests by sex. (D) Ct values of positive tests by swab type. ****, *P* < 0.0001, *t* test.

We also examined factors correlated with and affecting MPXV cycle threshold (Ct) values. Among all positive tests, Ct values at ARUP had an average of 23.81 (median 22.5), 1.54 cycles lower than Ct values at UW (mean 25.40, median 24.0), corresponding to approximately 2.9-fold more concentrated viral DNA following extraction assuming similar amplification efficiencies (Fig. 2A, *P* = 2.9 × 10^−6^, Welch’s *t* test). When Ct values of positive tests were examined by age, including only age groups (18 to 29, 30 to 39, 40 to 49, 50 to 59) with more than 30 results, the distribution was significantly different by age (*P* = 0.0408, ANOVA). When the distribution was visualized in a violin plot (Fig. 2B), the mode of the lower Ct peak remained unchanged (18 for ARUP, 22 for UW), however, the higher Ct peak, which was most apparent in the ARUP samples, became far less prominent with patient age. Ct values were the same for males and females at each institution (both: *P* > 0.9, Welch’s *t* test) (Fig. 2C). Among ARUP positive samples, metadata included the type of swab used (dry then submerged in PBS upon arrival at the lab, or in viral transport media [VTM]). Ct values of dry swabs were 1.65 cycles lower on average than swabs in VTM (22.79 versus 24.44, *P* = 5.26 × 10^−8^, Welch’s *t* test), or approximately 3.13 times fold more concentrated following extraction of viral DNA (Fig. 2D). Comparing only positive VTM swabs, Ct values at ARUP were still significantly less than those at UW (24.44 versus 25.40, *P* = 0.005, Welch’s *t* test).

### High concordance of multiple swabs sent from the same individual.

**FIG 3:**
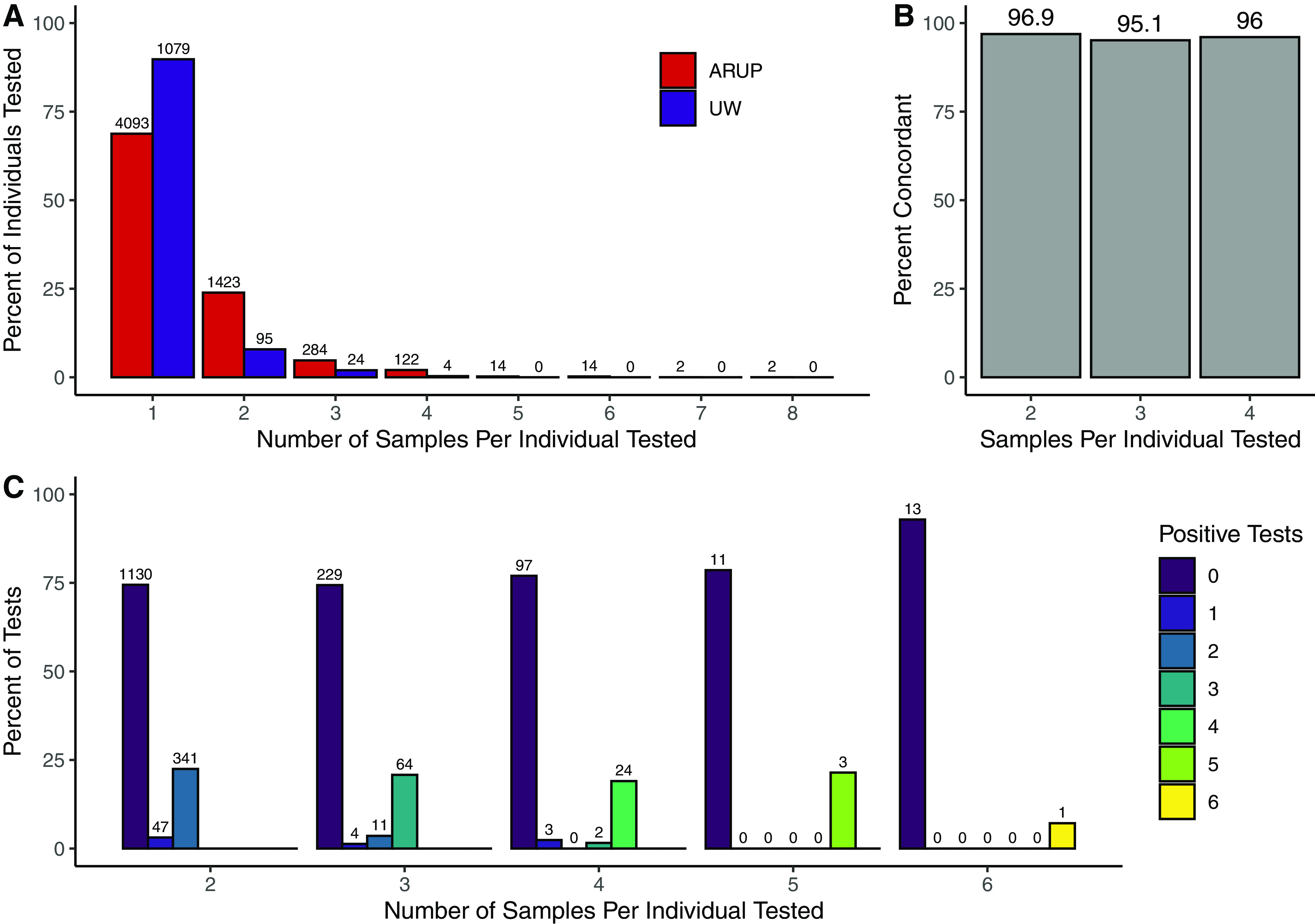
Examination of the yield and discordant results for individuals with multiple tests taken on the same collection date. (A) Percent of all individuals by number of replicate tests. Raw numbers are shown above bars. (B) Among replicate tests, percentage of individuals receiving all positive or all negative (concordant) test results. Percent is shown above bars. (C) For each group of n replicate samples per individuals, the percentage of individuals receiving 0, 1, or more positive tests. Raw numbers are shown above bars (e.g., two individuals had three positives and one negative results out of the 126 total individuals who had four swabs taken).

We have observed wide variation in the number of samples submitted for testing for each individual. While the majority of individuals had only a single sample submitted for testing, more than 31% of ARUP patients (*n* = 1,861) and more than 10% of UW patients (*n* = 123) had two or more specimens collected at the same time (Fig. 3A, data were filtered to exclude tests not collected on the same day). To explore the value of testing multiple swabs from the same patient, we examined test result concordance, defined as all results returning positive or all results returning negative. Among individuals with two, three, or four replicate samples submitted for testing, concordance was 96.9% when two swabs were submitted (*n* = 1,518), 95.1% when three swabs were submitted (*n* = 308), and 96.0% when four swabs were submitted (*n* = 126) (Fig. 3B). Notably, among individuals that received three or four samples tested, only 1.3% and 2.4% had a single positive test (Fig. 3C).

**FIG 4:**
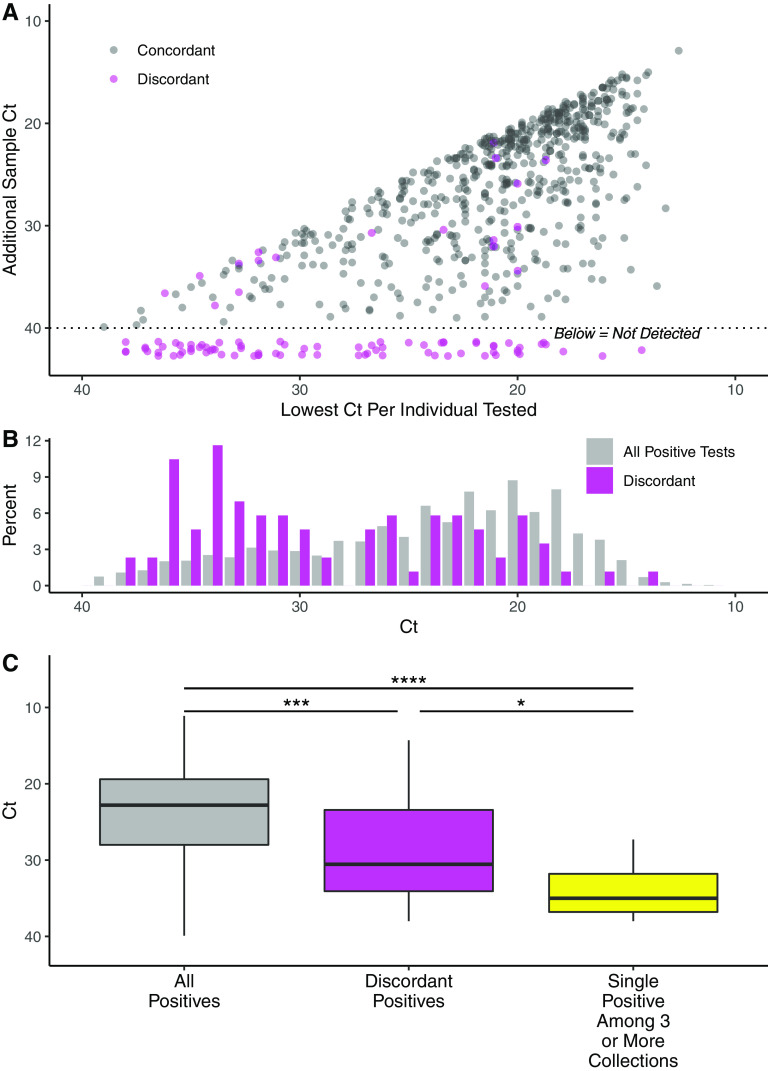
Relationship between Ct values and discordant results. (A) Comparison of Ct values for individuals that had two or more replicate specimens collected, and at least one positive result. The lowest Ct value is plotted on the *x* axis, and additional results plotted on the Y axis. Negative results are presented as higher than Ct 40. (B) Distribution of Ct values for individuals with discordant results (at least one negative and one positive; pink) versus all individuals with positive tests (gray). (C) Median and interquartile range of Ct values of positive results among all samples, among positive samples with at least one paired negative sample (discordant positives), and among samples that were the single positive test result when 2 or more additional replicate samples tested negative. *, *P* < 0.05; ***, *P* < 0.001; ****, *P* < 0.0001.

We speculated that individuals with discordant results may have lower viral burden and correspondingly higher Ct values. Fig. 4A shows a scatterplot of paired Ct values among individuals with at least one positive test result, with specimens from individuals with discordant results highlighted. The lowest Ct is plotted on the *x* axis and any matched Ct value is plotted on the *y* axis, with negative results plotted at Ct > 40. A strong positive relationship was observed (Pearson coefficient = 0.689), indicating that in most cases a low Ct value in one sample is likely to be repeated in a paired sample. Nevertheless, there exist a considerable number of concordant results with a large difference in Ct value between the two positive swabs. When only the Ct values from individuals with discordant results are plotted, a skew toward higher Ct values relative to all positive samples can be seen (Fig. 4B, magenta versus gray). Among all positive tests, only 18.2% of Ct values are higher than 30, while among discordant tests, 50.0% of Ct values are higher than 30. This is made clearer in Fig. 4C, which shows the median Ct for all positive tests (22.9), positive tests from individuals with discordant results (30.7), and single positive tests from individuals with three or four samples collected (35.0) (*P* < 2e^−16^, ANOVA).

## DISCUSSION

Here, we detail approximately 8 weeks of MPXV PCR testing data from July and August 2022 from two large reference laboratories. Our report substantially adds to the data available on monkeypox testing in the United States, as the main prior testing report from the CDC detailed testing of 2,009 specimens over a 6-week period from late May to June 2022 (2). Overall, testing was enriched in the population at greatest risk for monkeypox (males ages 18 to 40). We found a decline in positivity rate over time as more specimens were submitted, though positivity rates persisted at relatively high levels (~20%). We found a substantial difference in positivity rates between specimens submitted from males over that of females, consistent with prior reports of the vast majority of infections occurring in men who have sex with men (11).

The viral load of positive specimens was comparable between ARUP and UW, with a trend toward higher viral loads at ARUP. We hypothesize that viral load differences are due to extraction protocols, as UW extracts half the material of ARUP in order to add lysis buffer in a biosafety cabinet prior to use of extraction instrumentation. In addition, ARUP uses the Chemagic nucleic acid extractor which was previously associated with better analytical sensitivities for COVID-19 PCR (12). Unsurprisingly, dry swabs were associated with stronger viral loads than swabs in VTM because they are resuspended 6-fold less liquid and thus are more concentrated. Nonetheless, the workflow limitations to dry swab testing restrict the ability to test these specimens in a high-volume reference laboratory. Overall, although significant, differences in Ct values between institutions and swab types were very small and do not affect interpretation of test results.

Interestingly, we also noted small but significant differences in Ct values by age, which appeared to be driven by changes in Ct distribution in older versus younger individuals: A bimodal distribution of Ct values, with local maxima at Ct ~18 and ~31 in ARUP samples, was seen in 18- to 29-year-olds, with the peak at Ct 31 disappearing in 50- to 59-year-olds despite the mode at Ct 18 staying constant. We could not find any factors that confounded this analysis. Therefore, we speculate that immune factors may play a role in differences in distribution. One possibility is that age-related immune function decline leads to poorer control of MPXV, resulting in an “all or nothing” scenario, where MPXV infection leads to higher viral loads in older adults, whereas younger adults may sometimes control infection more effectively, resulting in lower viral loads and correspondingly higher Ct values. However, we also saw an effect in 50- to 59-year-olds, younger than is generally considered to be undergoing immune senescence (13). A second possibility that explains the distribution is that preexisting immunity due to vaccinia immunization may allow clearance of lower viral load MPXV infections. As routine inoculation continued in the United States until 1972 to protect against smallpox infection, we expect that most individuals aged 50 and older included in this study have been vaccinated against vaccinia. Additional studies have found 9% (11) or 13% (14) of individuals with MPXV infection report prior smallpox vaccination. However, these findings were not broken down by age, nor do they account for the effects of pre- and postexposure prophylactic vaccination, which in the United States began in July 2022. Unfortunately, our limited access to clinical metadata prevents further examination of viral loads as a function of vaccination status, and to our knowledge, no published studies examine this question. Clearly, study of the effect of varicella vaccination on MPXV viral load and clearance kinetics is merited.

As CDC recommendations encourage collecting two swabs, we specifically examined our testing data to understand the utility of submitting multiple lesion swabs for detection of MPXV. Skin lesions have already shown the highest sensitivity (15) and submitting multiple specimens in separate tubes increases the labor demands on clinic and laboratory staff and may substantially increase costs associated with sending MPXV testing. While the vast majority of individuals had only one specimen submitted, approximately one-third of individuals tested at ARUP had multiple specimens submitted, suggesting a considerable portion of MPXV PCR testing is associated with this use case. Testing results for multiple specimens were highly concordant: Among individuals with precisely two samples submitted from a single testing event, 96.7% were concordant, and we found only 1.5% of individuals with three or more swabs submitted had only one swab test positive. Discordant positives were also associated with substantially lower viral loads than the total positive cohort. These discordant results may be explained by one of two major causes: first, these samples may have contained a low viral load with stochasticity near the limit of detection affecting sensitivity. Alternatively, discordant samples may be false positives resulting from cross-contamination by an adjacent high viral load specimen. On August 23, 2022 the CDC issued a Lab Advisory warning of false-positive risk and recommended repeat testing of samples with Cts greater than 34 (16). However, a specimen which repeated as negative does not rule out stochastic effects for samples near the limit of detection. Overall, our data suggest current CDC recommendations of sampling at least two lesions per patient provides sufficient diagnostic performance for the vast majority of patients, given the importance of not missing MPXV infections (17). With the availability of VTM testing now, we believe the most practical method for specimen collection is to combine swabs into a single tube of VTM when collected from the same source in order to reduce overall testing costs, though this may subtly impact potential determination of false positives via discordant results.

Limitations of this work include its descriptive nature, restriction to lesion swabs, and use of reference laboratory data with sparse clinical metadata. We did not specifically consider diagnostic yield in nonstandard specimen types as data for these specimens was very sparse. Furthermore, determining the utility of specific specimen types is best performed in the context of prospective studies, although retrospective, descriptive examinations of clinical testing data can generate hypotheses for how diagnostic testing is being used in the real world. In particular, positivity rates are highly influenced by choices made by providers performing testing. For those few individuals who had discordant results associated with low viral loads, we are unable to draw on additional or orthogonal testing data or electronic health record data to adjudicate the likelihood of a false positive (18). Nor were we able to determine if any asymptomatic individuals tested positive, as has been recently reported (19). The availability of MPXV serology would help adjudicate these cases; however, currently no authorized commercial MPXV serological assays nor fully MPXV-specific assays exist. It is likely more work is needed on transitioning accurate MPXV serology testing from CDC to academic and clinical reference laboratories in a similar manner as the MPXV PCR testing described here.
